# miR-125a-5p inhibits tumorigenesis in hepatocellular carcinoma

**DOI:** 10.18632/aging.102276

**Published:** 2019-09-17

**Authors:** Xin Xu, Yuquan Tao, Yongjie Niu, Zhixian Wang, Congcong Zhang, Yongchun Yu, Lifang Ma

**Affiliations:** 1 Shanghai Municipal Hospital of Traditional Chinese Medicine, Shanghai University of Traditional Chinese Medicine, Shanghai 200071, P.R. China; 2 Institute for Thoracic Oncology, Shanghai Chest Hospital, Shanghai Jiao Tong University, Shanghai 200030, P.R. China; 3 Department of Clinical Laboratory Medicine, Shanghai Municipal Hospital of Traditional Chinese Medicine, Shanghai University of Traditional Chinese Medicine, Shanghai 200071, P.R. China

**Keywords:** miR-125a-5p, HCC, PTPN1, MAP3K11, MAPK signaling pathway

## Abstract

Hepatocellular carcinoma (HCC) is one of the most prevalent and deadly cancers world-wide. miR-125a-5p is a tumor suppressor in HCC and other cancers, but its mechanisms of action during HCC tumorigenesis remain largely unknown. In this study, we found that miR-125a-5p expression was significantly lower in HCC tissues and cell lines than matched normal tissues and liver cells. miR-125a-5p overexpression inhibited HCC cell proliferation and induced apoptosis *in vitro* and *in vivo*, while miR-125a-5p knockdown had the opposite effects. In addition, PTPN1 and MAP3K11 were identified as targets of miR-125a-5p. Knocking down PTPN1 and MAP3K11 activated the JNK MAPK signaling pathway to suppress HCC cell proliferation and induce apoptosis. Our findings suggest that miR-125a-5p may be a useful therapeutic target for treatment of HCC patients.

## INTRODUCTION

Hepatocellular carcinoma (HCC) was the sixth most common type of malignant tumor and the fourth leading cause of cancer-related death worldwide in 2018 [1]. An estimated 80-90% of HCC cases are caused by cirrhosis resulting from chronic hepatitis B (HBV) or hepatitis C (HCV) infections [2]. Chronic HBV/HCV infections, alcoholic liver disease, metabolic syndromes, and some rare autoimmune or genetic conditions may also be risk factors for HCC [3, 4]. Even though treatments for HCC, including surgical resection, chemotherapeutic regimens, liver transplantation, local ablation, and targeted therapy, have greatly improved over the past few decades, the survival rates remain low for HCC patients [5]. A better understanding of the molecular mechanisms underlying the development and progression of HCC might help identify potential therapeutic targets and improve clinical outcomes.

microRNAs (miRNAs), a type of non-coding RNA, are about 23 nucleotides in length and act as important gene regulators by repressing translation or degrading RNA in animals and plants [6, 7]. miRNAs control the expression of their target mRNAs mainly by binding to 3'-untranslated regions (3′-UTRs) [8]. miRNAs can promote or inhibit cell proliferation, apoptosis, metastasis, and angiogenesis, and abnormal miRNA expression is associated with many cancers and other diseases [9]. Recent findings indicate that miRNAs might act as potential diagnostic and prognostic biomarkers and as therapeutic targets in HCC depending on their biological characteristics and functions [10–12].

Many studies have demonstrated that miR-125a-5p, a tumor suppressor, is down-regulated in cancers and participates in cancer development and progression by regulating cell proliferation, apoptosis, migration, invasion, and metastasis. For example, in lung cancer, miR-125a-5p inhibits cancer cell proliferation, migration, and invasion and induces apoptosis by targeting Suv39H1 and p53 [13, 14]. miR-125a-5p also inhibits cell proliferation and induces apoptosis in colon cancer by targeting BCL2, BCL2L12, and Mcl-1 [15]. In HCC, miR-125a-5p suppresses cell proliferation, migration, and angiogenesis by regulating SIRT7, VEGF, MMP11, c-RAf, and ErbB3 [16–19]. miR-125a-5p is also involved in breast, gastric, cervical, prostate, and bladder cancer [20–24]. However, the role of miR-125a-5p in HCC tumorigenesis remains largely unknown.

Protein tyrosine phosphatase N1 (PTPN1), also known as protein tyrosine phosphatase 1B (PTP1B), is a member of the protein tyrosine phosphatase family. The role of PTPN1 in cancer is controversial, as it has been shown to act as both a tumor suppressor and a tumor promoter [25]. In B-cell lymphoma and esophageal cancer, it is a tumor suppressor [26, 27]. In contrast, PTPN1 is as a tumor promoter in colorectal, non-small cell lung, breast, gastric, and colon cancer [28–33]. The role of PTPN1 in HCC remains unknown.

Mitogen activated protein kinase kinasekinase 11 (MAP3K11), also called mixed lineage kinase 3 (MLK3), is a member of the mixed lineage kinase subfamily of serine/threonine kinases. MAP3K11 is an oncogene that promotes tumorigenesis in breast and colorectal cancer [34–37]. Whether MAP3K11 also plays a role in HCC has not yet been investigated.

In this study, we investigated the role of miR-125a-5p in HCC and whether PTPN1 and MAP3K11 are involved in its effects.

## RESULTS

### miR-125a-5p is down-regulated in HCC tissues and cell lines

**Figure 1:**
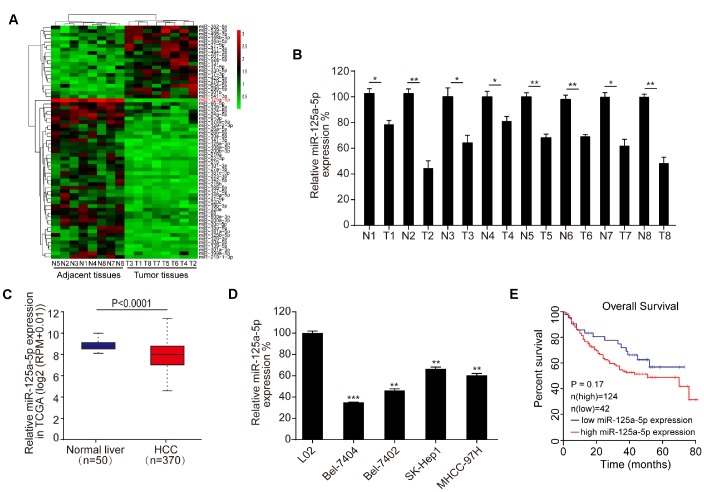
**miR-125a-5p is down-regulated in HCC tissues and cells.** (**A**) Heat map of differentially expressed miRNAs in eight matched pairs of HCC tissues and adjacent normal tissues. (**B**) qRT-PCR indicated that miR-125a-5p expression was down-regulated in HCC tissues compared to the adjacent normal tissues. Student’s t-test, mean ± SD, *P<0.05, **P<0.01. (**C**) miR-125a-5p expression was down-regulated in HCC tumor tissues compared to adjacent normal tissues in TCGA in Starbase. (**D**) qRT-PCR indicated that miR-125a-5p expression was lower in HCC cell lines (Bel-7404, SK-Hep1, Bel-7404, and MHCC-97H) than in a normal liver cell line (L02). Student’s t-test, mean ± SD, **P<0.01, ***P<0.001. (**E**) Kaplan-Meier curves indicated that overall survival of HCC patients was not associated with miR-125a-5p expression level in Kaplan Meier Plotter.

We used a microarray to compare miRNA expression in eight matched pairs of HCC tissues and adjacent normal tissues. Differentially expressed miRNAs, which were defined by a ≥ 2-fold change in expression between the two tissue types and associated P-values ≤ 0.05, are shown in the heat map in Figure 1A. The microarray assay indicated that miR-125a-5p expression was decreased in HCC tissues compared to adjacent normal tissues; this was also the most statistically significant difference observed (Figure 1A, Supplementary Table 1). qRT-PCR also revealed that miR-125a-5p expression was down-regulated in the eight matched pairs of HCC tissues compared to the adjacent normal tissues (Figure 1B). Moreover, miR-125a-5p expression was down-regulated in HCC tumor tissues compared to adjacent normal tissues in TCGA from the Starbase database [38] (http://starbase.sysu.edu.cn/) (Figure 1C). In addition, qRT-PCR indicated that miR-125a-5p expression was lower in HCC cell lines (Bel-7404, SK-Hep1, Bel-7404, and MHCC-97H) than in a normal liver cell line (L02) (Figure 1D). However, Kaplan-Meier survival curves indicated that overall survival in HCC patients was not associated with miR-125a-5p expression level [39] (http://www.kmplot.com/mirpower) (Figure 1E). This may be due to the small number of samples examined. Furthermore, there is a particularly large amount of heterogeneity among HCC patients which might also have prevented detection of associations between miR-125a-5p expression and survival.

### miR-125a-5p suppresses proliferation and induces apoptosis in HCC cells *in vitro*

**Figure 2:**
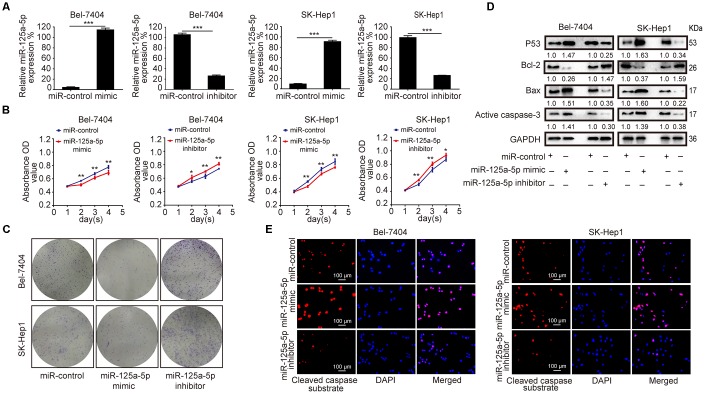
**miR-125a-5p suppresses proliferation and induces apoptosis in HCC cells *in vitro*.** (**A**) qRT-PCR showed that miR-125a-5p was up-regulated after transfection with mimics and down-regulated after transfection with inhibitors in Bel-7404 and SK-Hep1 cells. Student’s t-test, mean ± SD, ***P<0.001. (**B** and **C**) Cell proliferation ability was assessed using CCK8 and colony formation assays in transfected Bel-7404 and SK-Hep1 cells. Two-way ANOVA, mean ± SD, *P<0.05, **P<0.01. (**D**) Western blots were used to analyze the expression of p53, Bax, Bcl-2, and active caspase-3 after transfection in Bel-7404 and SK-Hep1 cells. (**E**) Immunofluorescence staining of cleaved caspase substrate was detected after transfection in Bel-7404 and SK-Hep1 cells. Scale bars: 100μm.

To further investigate its biological function in HCC, miR-125a-5p was overexpressed and knocked down in Bel-7404 and SK-Hep1 cells by transfection with miR-125a-5p mimics and inhibitors, respectively. The qRT-PCR assay showed that miR-125a-5p was up-regulated 24 hours after transfection with mimics and down-regulated 24 hours after transfection with inhibitors in both cell lines (Figure 2A, Supplementary Figure 1A). CCK8 and colony formation assays revealed that overexpression and knockdown of miR-125a-5p suppressed and enhanced cell proliferation, respectively (Figure 2B and 2C, Supplementary Figure 1B). P53, Bax, Bcl-2, and caspase-3 are the classical biomarkers associated with cell apoptosis. Western blots revealed that miR-125a-5p overexpression increased p53, Bax, and active caspase-3 expression, but reduced Bcl-2 expression. Conversely, miR-125a-5p knockdown had the opposite effects (Figure 2D, Supplementary Figure 1C). In addition, immunofluorescence (IF) assays showed that miR-125a-5p overexpression increased, while miR-125a-5p knockdown reduced, cleaved caspase substrate levels (Figure 2E, Supplementary Figure 1D). miR-125a-5p therefore suppresses proliferation and induces apoptosis in HCC cells *in vitro*.

### PTPN1 and MAP3K11 are direct targets of miR-125a-5p in HCC

**Figure 3:**
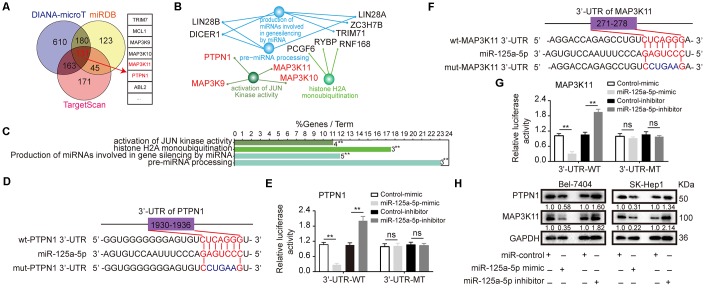
**PTPN1 and MAP3K11 are direct targets of miR-125a-5p in HCC.** (**A**) Three target prediction databases were used to predict the targets of miR-125a-5p; 131 common elements were identified. (**B** and **C**) These 131 target genes were analyzed with ClueGO and CluePedia in Cytoscape. (**D** and **F**) miR-125a-5p and its putative binding sequences in the 3′-UTRs of PTPN1 and MAP3K11. (**E** and **G**) miR-125a-5p overexpression inhibited, while knockdown increased, luciferase activity of the wild-type (WT), but not mutant (MT), PTPN1 or MAP3K11 3′-UTRs. Student’s t-test, mean ± SD, **P<0.01, ns: not statistically significant. (**H**) PTPN1 and MAP3K11 protein expression was assessed via Western blot in transfected Bel-7404 and SK-Hep1 cells.

To predict the target genes of miR-125a-5p, we searched the TargetScan [40] (http://www.targetscan.org/), miRDB [41] (http://www.mirdb.org/), and DIANA-microT [42] (http://www.microrna.gr/webServer) databases; 131 common elements were identified among the databases (Figure 3A, Supplementary Table 2). We then analyzed the biological functions and pathways of these common elements using ClueGO [43] (http://www.ici.upmc.fr/cluego/cluegoDownload.shtml) and CluePedia [44] (http://www.ici.upmc.fr/cluepedia/) in Cytoscape [45] (http://www.cytoscape.org/). The results indicated that “activation of JUN kinase activity” is a biological function of miR-125a-5p and that PTPN1, MAP3K9, MAP3K10 and MAP3K11 are potential targets of this miRNA (Figure 3B and 3C, Supplementary Figure 2A). However, the predicted effects for MAP3K9 were inconsistent; Starbase indicated that it was up-regulated, while GEPIA indicated that it was down-regulated (Supplementary Figure 2B). That gene was therefore excluded from further analysis. Kaplan-Meier survival curves indicated that overall survival in HCC patients was not associated with MAP3K10 expression level in the Kaplan Meier Plotter and GEPIA databases (Supplementary Figure 2C). We therefore focused on PTPN1 and MAP3K11 for the remainder of the study because their functions in HCC were not clear after database analysis.

Next, we found that the 3′-UTR sequences of PTPN1 and MAP3K11 matched the “seed sequence” of miR-125a-5p, indicating that it targets both genes (Figure 3D and 3F). Moreover, a dual-luciferase reporter assay indicated that miR-125a-5p overexpression inhibited, while miR-125a-5p knockdown increased, luciferase activity of the wild-type (WT), but not the mutant (MT), PTPN1 or MAP3K11 3′-UTRs (Figure 3E and 3G). In addition, miR-125a-5p overexpression significantly inhibited PTPN1 and MAP3K11 mRNA and protein expression in Bel-7404 and SK-Hep1 cells (Figure 3H, Supplementary Figure 2D and 2E). Moreover, miR-125a-5p knockdown increased PTPN1 and MAP3K11 protein and mRNA expression in Bel-7404 and SK-Hep1 cells (Figure 3H, Supplementary Figure 2D and 2E). These results indicate that PTPN1 and MAP3K11 are direct targets of miR-125a-5p.

### PTPN1 and MAP3K11 are up-regulated and negatively correlated with miR-125a-5p expression in HCC

**Figure 4:**
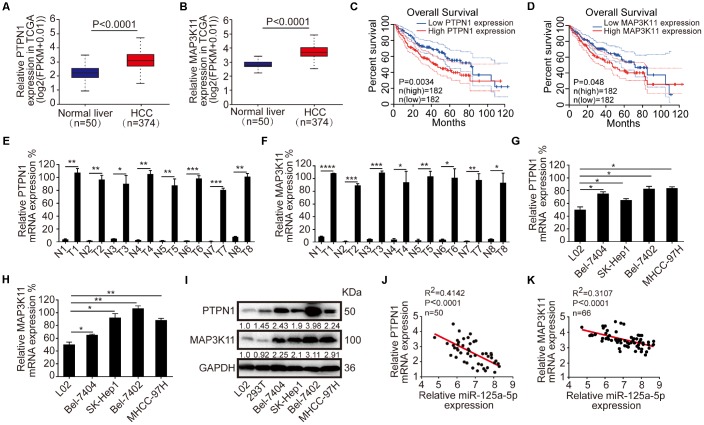
**PTPN1 and MAP3K11 are up-regulated and negatively correlated with the expression of miR-125a-5p in HCC.** (**A** and **B**) PTPN1 and MAP3K11 were up-regulated in TCGA in Starbase. (**C** and **D**) High expression of PTPN1 and MAP3K11 was associated with poorer overall survival in the GEPIA database. (**E** and **F**) qRT-PCR indicated that PTPN1 and MAP3K11 were up-regulated in the eight matched pairs of HCC tissues compared to the adjacent normal tissues. Student’s t-test, mean ± SD, *P<0.05, **P<0.01, ***P<0.001, ****P<0.0001. (**G**–**I**) qRT-PCR and Western blots indicated that PTPN1 and MAP3K11 were up-regulated in HCC cell lines. One-way ANOVA, mean ± SD, *P<0.05, **P<0.01. (**J** and **K**) Spearman correlation analysis showed that miR-125a-5p expression was negatively correlated with PTPN1 and MAP3K11 expression.

Next, we analyzed PTPN1 and MAP3K11 expression in HCC. Both genes were up-regulated in HCC according to the Starbase database [38] (http://starbase.sysu.edu.cn/) (Figure 4A and 4B), and high PTPN1 and MAP3K11 expression was associated with poorer overall survival in the GEPIA database [46] (http://gepia.cancer-pku.cn/) (Figure 4C and 4D). qRT-PCR revealed that PTPN1 and MAP3K11 were up-regulated in the eight HCC tissue samples compared to the adjacent normal tissues (Figure 4E and 4F). qRT-PCR and Western blot indicated that PTPN1 and MAP3K11 also were up-regulated in HCC cell lines (Figure 4G–4I, Supplementary Figure 3A). Moreover, Spearman correlation analysis indicated that miR-125a-5p expression was negatively correlated with PTPN1 and MAP3K11 expression (Figure 4J and 4K). Finally, knockdown of PTPN1 and MAP3K11 resulted in reduced PTPN1 and MAP3K11 protein expression (Supplementary Figure 3B and 3C). Together, these results suggest that PTPN1 and MAP3K11 are up-regulated and negatively correlated with miR-125a-5p expression in HCC.

### miR-125a-5p suppresses PTPN1 and MAP3K11 expression via the MAPK signaling pathway in HCC

**Figure 5:**
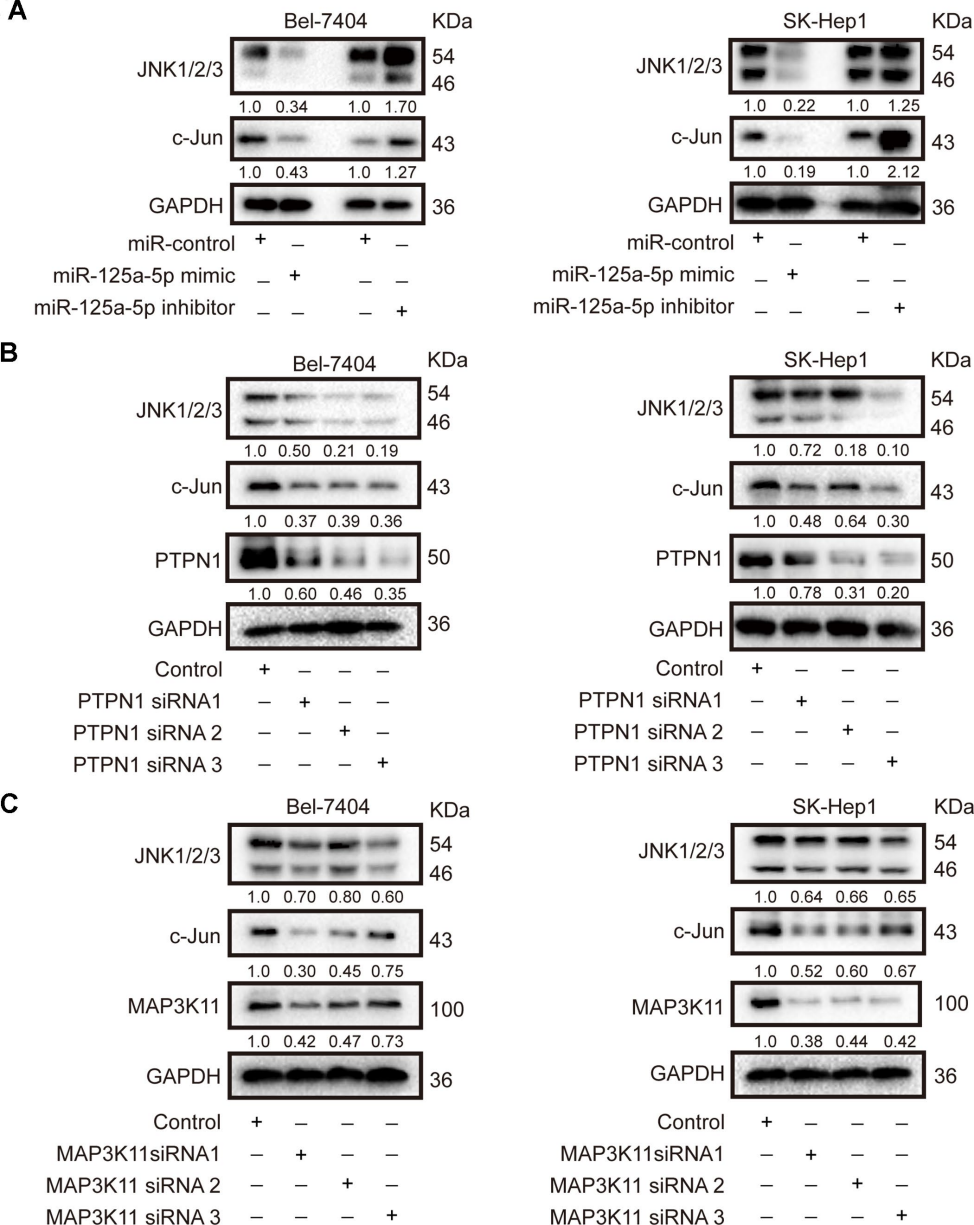
**miR-125a-5p suppresses PTPN1 and MAP3K11 expression via the MAPK signaling pathway in HCC.** (**A**) Western blots indicated that miR-125a-5p overexpression reduced, while miR-125a-5p knockdown increased, the expression of JNK1/2/3 and c-Jun in Bel-7404 and SK-Hep1 cells. (**B** and **C**) Western blots showed that PTPN1 and MAP3K11 knockdown decreased the expression of JNK1/2/3 and c-Jun in Bel-7404 and SK-Hep1 cells.

In mammals, there are four major subgroups of the MAPK family: the extracellular signal-regulated kinases 1 and 2 (ERK1/2), JNK, p38, and ERK5 pathways [47]. As mentioned above, “activation of JUN kinase activity” was one of the biological functions associated with the 131 common elements identified using target prediction databases, implying that the JNK MAPK signaling pathway might be essential for the biological function of miR-125a-5p in HCC. Next, we confirmed that miR-125a-5p overexpression reduced, while miR-125a-5p knockdown increased, the expression of JNK1/2/3 and c-Jun in Bel-7404 and SK-Hep1 cells (Figure 5A, Supplementary Figure 4A). Knockdown of PTPN1 and MAP3K11 also decreased JNK1/2/3 and c- Jun expression in Bel-7404 and SK-Hep1 cells (Figure 5B and 5C, Supplementary Figure 4B and 4C). These results indicate that miR-125a-5p suppressed PTPN1 and MAP3K11 expression via the MAPK signaling pathway in HCC.

### miR-125a-5p suppresses cell proliferation and induces apoptosis in HCC by targeting PTPN1 and MAP3K11 via the MAPK signaling pathway

**Figure 6:**
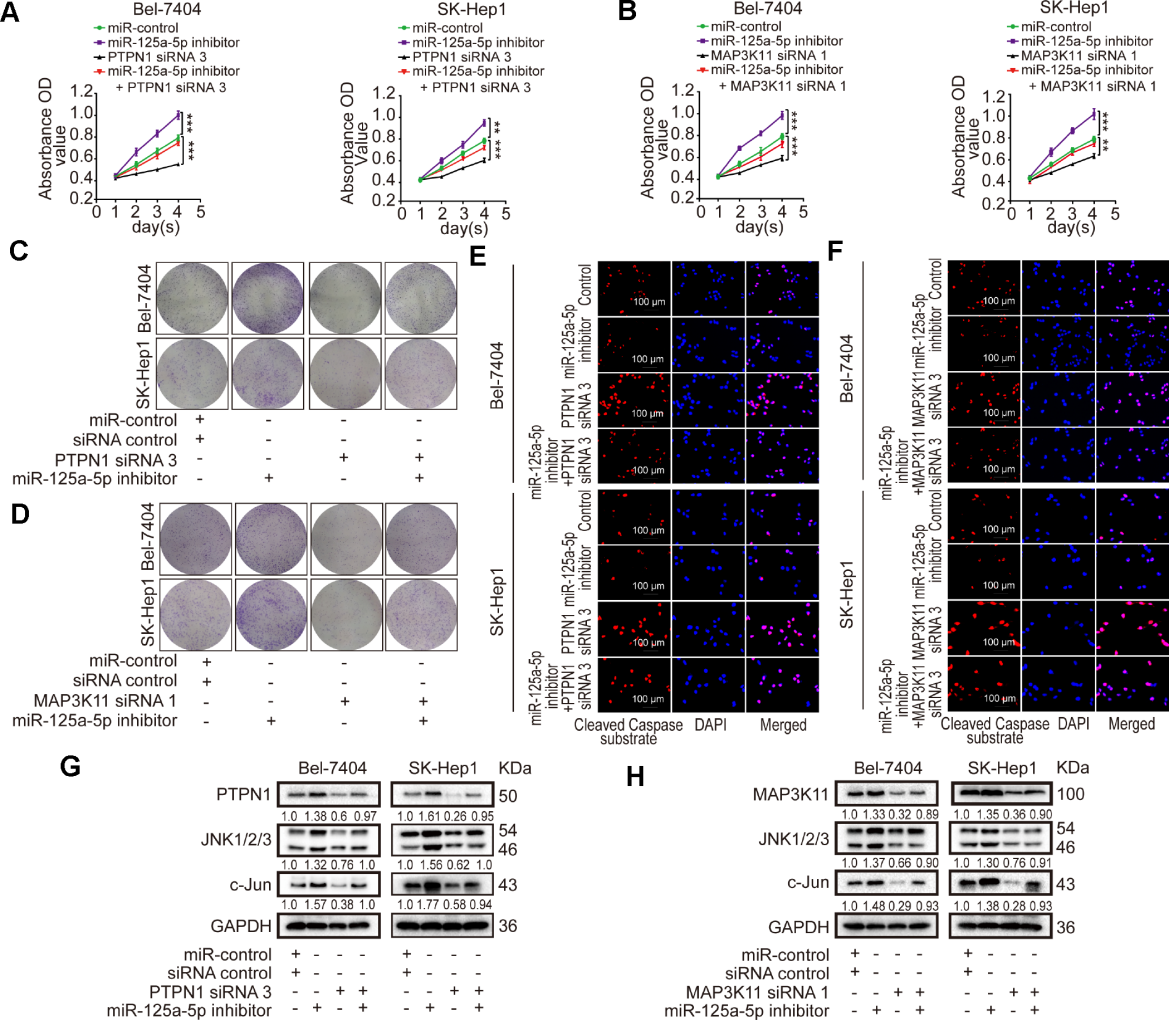
**miR-125a-5p suppresses cell proliferation and induces apoptosis in HCC by targeting PTPN1 and MAP3K11 via the MAPK signaling pathway.** (**A**–**D**) Cell proliferation ability was assessed using CCK8 and colony formation assays after Bel-7404 and SK-Hep1 cells were transfected with miR-control, miR-125a-5p inhibitors, PTPN1 siRNA 3, or MAP3K11 siRNA 1. Two-way ANOVA, mean ± SD, **P<0.01, ***P<0.001. (**E** and **F**) Immunofluorescence staining of cleaved caspase substrate was detected after transfection in Bel-7404 and SK-Hep1 cells. Scale bars: 100μm. (**G** and **H**) Expression of PTPN1, MAP3K11, JNK1/2/3, and c-Jun was examined in transfected Bel-7404 and SK-Hep1 cells using Western blots.

Loss of function experiments were performed using PTPN1 siRNA 3 and MAP3K11 siRNA 1, which had the highest knockdown efficiencies for their targets. The loss-of-function experiments included four groups: a control group, a group transfected with miR-125a-5p inhibitors, a group transfected with PTPN1 siRNA 3 or MAP3K11 siRNA 1, and a group co-transfected with miR-125a-5p inhibitors and PTPN1 siRNA 3 or MAP3K11 siRNA 1. CCK8 and colony formation assays showed that cell proliferation was increased in the miR-125a-5p inhibitors group and suppressed in the PTPN1 or MAP3K11 group. Immunofluorescence assays revealed that cell apoptosis was suppressed in the miR-125a-5p inhibitors group and upregulated in the PTPN1 or MAP3K11 group. Cell proliferation and apoptosis rates were similar in the co-transfected and control groups (Figure 6A–6F, Supplementary Figure 5A and 5B). In addition, co-transfection restored PTPN1, MAP3K11, JNK1/2/3, and c-Jun protein expression to control levels (Figure 6G and 6H, Supplementary Figure 5C). In addition, we performed a gain of function assay by inserting PTPN1 and MAP3K11 CDS into pCDNA3.1 (+) vectors. Western blot assays confirmed acceptable rescue efficiency and ensured that the siRNAs had no off-target effects (Supplementary Figure 5D). Together, these results indicate that miR-125a-5p suppresses cell proliferation and induces apoptosis in HCC by targeting PTPN1 and MAP3K11 via the MAPK signaling pathway.

### miR-125a-5p suppresses cell proliferation and induces apoptosis in HCC by targeting PTPN1 and MAP3K11 via the MAPK signaling pathway *in vivo*

**Figure 7:**
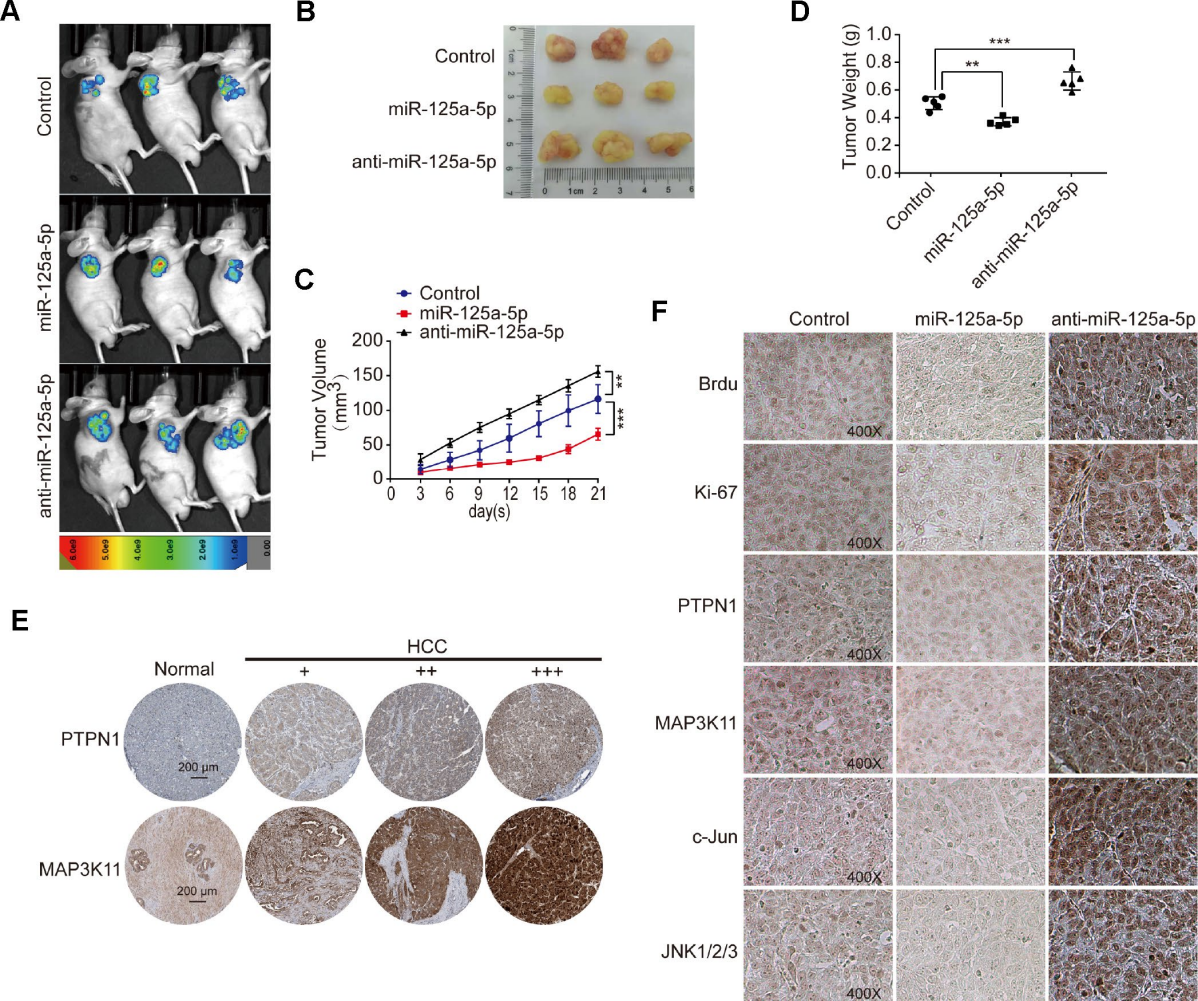
**miR-125a-5p suppresses cell proliferation and induces apoptosis by targeting PTPN1 and MAP3K11 via the MAPK signaling pathway *in vivo*.** (**A** and **B**) Representative images of mice and xenografted tissues. (**C** and **D**) Xenograft tissue volumes and weights were analyzed (n=5). Two- and One-way ANOVAs, mean ± SD. **P<0.01, ***P<0.001. (**E**) PTPN1 and MAP3K11 expression was higher in HCC tumor tissues than in normal liver tissues in the Human Protein Atlas database. (**F**) Brdu, Ki67, PTPN1, MAP3K11, JNK1/2/3, and c-Jun expression were detected via immunofluorescence in xenografted tissuesat 400× magnification.

We established a subcutaneous tumor model in mice and removed the xenografted tissues after images were taken. Representative images of the mice and xenografted tissues are shown in Figure 7A and 7B. Analysis of the xenografted tissue volumes and weights revealed that miR-125a-5p overexpression suppressed, while miR-125a-5p knockdown promoted, tumor growth (Figure 7C and 7D). Moreover, PTPN1 and MAP3K11 expression was higher in HCC tumor tissues than in normal liver tissue in the Human Protein Atlas database [48] (http://www.proteinatlas.org) (Figure 7E). A Chi-square test indicated that PTPN1 and MAP3K11 expression was also higher in HCC tissues than normal liver tissues in human liver cancer tissue microarray(TMA) slides purchased from U.S. Biomax (NO: LV1501) containing 10 normal liver and 120 HCC tissues (Supplementary Tables 3–5). Brdu and Ki67 staining of the xenograft tissues indicated that miR-125a-5p overexpression decreased the number of proliferating cells and increased the number of apoptotic cells, while miR-125a-5p knockdown had the opposite effects (Figure 7F). In addition, miR-125a-5p overexpression decreased PTPN1, MAP3K11, JNK1/2/3, and c-Jun expression, while miR-125a-5p knockdown had the opposite effects (Figure 7F). Taken together, these results demonstrate that miR-125a-5p may suppress cell proliferation and induce apoptosis in HCC by targeting PTPN1 and MAP3K11 via the MAPK signaling pathway.

## DISCUSSION

In this study, we found that miR-125a-5p was down-regulated in HCC tissues and cell lines and that it inhibited HCC cell proliferation and induced cell apoptosis both *in vitro* and *in vivo*. In addition, PTPN1 and MAP3K11 were identified as targets of miR-125a-5p. Furthermore, miR-125a-5p inhibited HCC cell proliferation and induced cell apoptosis by targeting PTPN1 and MAP3K11 via the MAPK signaling pathway.

Protein tyrosine phosphatase and tyrosine kinases modulate cellular levels of tyrosine phosphorylation and regulate cellular events such as proliferation, differentiation, and motility [49]. It is well established that the protein tyrosine phosphatase PTPN1 directly regulates the insulin and the leptin signaling pathway, making it a promising therapeutic target for type II diabetes and obesity [50]. However, PTPN1 plays conflicting roles in different types of tumors [26–33]. Here, we found that PTPN1 was a tumor promoter, and its expression increased in HCC tissues and cell lines. Moreover, high PTPN1 expression was associated with poorer overall survival in the GEPIA database.

MAP3K11 phosphorylates several MAP2Ks to activate c-Jun-N-terminal kinase (JNK) and downstream c-Jun transcription factors [51]. MAP3K11 can also activate p38 and help facilitate the activation of B-Raf, which stimulates the ERK 1/2 signaling pathway [52, 53]. Previous studies reported that MAP3K11 acts as an oncogene and participates in the tumorigenesis process in breast and colorectal cancer [34–37]. Here, we found that MAP3K11 expression was increased in HCC tissues and cell lines, and higher MAP3K11 was associated with poorer overall survival in the GEPIA database.

We also found that miR-125a-5p suppressed HCC cell proliferation and induced cell apoptosis by directly targeting PTPN1 and MAP3K11 via the JNK MAPK signaling pathway in HCC. The JNK pathway regulates many physiological processes, including inflammatory responses and cell proliferation, differentiation, and death [54]. The JNK pathway is activated by two upstream mitogen activated protein kinase kinases (MAP2Ks) that directly phosphorylate JNKs on threonine (Thr183) and tyrosine (Tyr185) residues [55]. In turn, MAP2Ks are activated by upstream pathways through mitogen activated protein kinase kinase kinases (MAP3Ks), such as MEKK-4, MLK2, and MLK3 [56]. Upon activation by the upstream MAP2Ks, JNKs phosphorylate and activate several nuclear and non-nuclear proteins downstream of JNKs, such as c-Jun, Jun B, Jun D, c-Myc, p53, and the Bcl-2 family [57]. These proteins control cellular proliferation, differentiation, and apoptosis [58].

MAP3K11, which is a MAP3K, is an upstream activator of JNKs, which in turn activate the downstream proteins c-Jun, p53, and the Bcl-2 family and ultimately result in phenotypic changes in HCC cells. We found that miR-125a-5p overexpression increased p53 and Bax expression and reduced Bcl-2 expression, while miR-125a-5p knockdown led had the opposite effects. PTPN1 also activated JNKs and had the same effects as MAP3K11. Once c-Jun is activated, it increases the activity of transcription factor activator protein-1 (AP-1) upon dimerization with Fosproteins [57]. This increases expression of apoptotic precursor factors such as NF-κB, CD95L, p53, and TNF-α, leading to cell apoptosis [59, 60]. Additionally, activated JNKs can promote apoptosis by directly inducing p53 activity [61–63]. JNKs also promote the activation of the Bcl-2 family in mitochondria; this induces the release of cytochrome C, which interacts with caspase-3 to cause apoptosis, into the cytoplasm [64, 65]. Here, we also found that miR-125a-5p overexpression increased, while miR-125a-5p knockdown reduced, cleaved caspase substrate and caspase-3 expression, implying that JNKs also directly act on mitochondria to activate caspase-3 and induce cell apoptosis.

**Figure 8:**
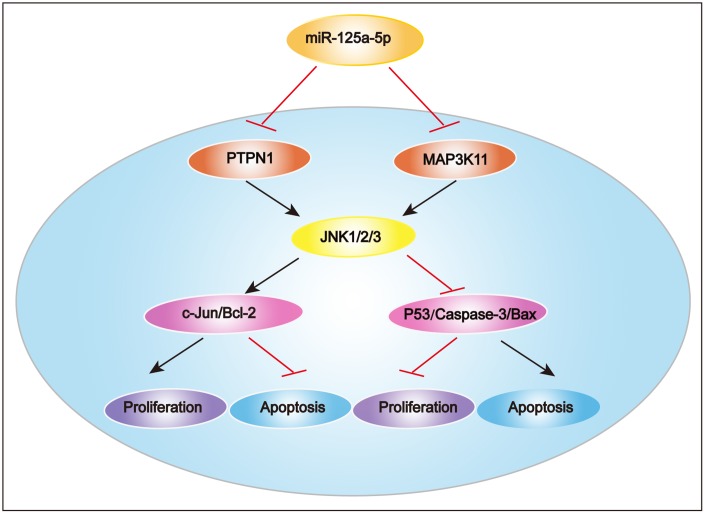
**Proposed mechanism of miR-125a-5p function in HCC.** miR-125a-5p inhibits HCC cell proliferation and induces cell apoptosis by directly targeting PTPN1 and MAP3K11 via the MAPK signaling pathway.

In summary, we found in this study that miR-125a-5p expression was significantly decreased in HCC tissues and cell lines compared to matched normal tissues and normal liver cells. miR-125a-5p overexpression inhibited HCC cell proliferation and induced apoptosis *in vitro* and *in vivo*, while miR-125a-5p knockdown had the opposite effects. Furthermore, PTPN1 and MAP3K11 were identified as targets of miR-125a-5p, and knockdown of PTPN1 and MAP3K11 inhibited the JNK signaling pathway to suppress HCC cell proliferation and induce apoptosis. These results indicate that miR-125a-5p inhibits HCC cell proliferation and induces cell apoptosis by directly targeting PTPN1 and MAP3K11 via the MAPK signaling pathway (Figure 8). miR-125a-5p mimics or JNK signaling inhibitors alone or in combination might therefore be potential treatments for HCC.

## MATERIALS AND METHODS

### HCC tissue specimens

Eight pairs of fresh HCC tissue and adjacent normal tissue samples were collected from patients with HCC in our previous study [66]. Patients with other comorbidities, such as liver cirrhosis or hepatitis, were excluded from that study; only those with primary liver cancer alone were included. The specimens were frozen immediately after surgery in liquid nitrogen. None of the patients received radiotherapy, chemotherapy, or other treatments before surgery. All patients provided written informed consent for use of the samples during their hospitalization. The informed consent documents were reviewed and approved by the Ethics Committee of Shanghai Municipal Hospital of Traditional Chinese Medicine.

### miRNA microarray

miRNA expression profiles in the HCC and matched control tissues were examined in a miRNA array (Shanghai Wcgene Biotech, Shanghai, China) using high-throughput quantitative RT-PCR (qRT-PCR). Total RNA was isolated from tissue samples, purified, polyadenylated in a poly (A) polymerase reaction, and then reversed-transcribed into cDNA. Individual miRNAs were quantified with specific MystiCq microRNA qPCR assay primers (Sigma, St. Louis, USA).

### Human liver cancer tissue microarray

Human liver cancer tissue microarray (TMA) slides were purchased from U.S. Biomax (Rockville, LV1501, MD, USA,). Slides were incubated in primary antibodies against PTPN1 (Abcam, ab75856, Cambridge, UK) and MAP3K11 (Abcam, ab51068, Cambridge, UK).

### Cell lines and cell culture

The human cell lines (Bel-7404, SK-Hep1, Bel-7402, MHCC-97H, L02, and 293T) used in this study were obtained from the American Type Culture Collection (ATCC, MD, USA). All cells were cultured in Dulbecco’s modified Eagle medium-high glucose (DMEM; Sigma, RNBG6034, St. Louis, USA) supplemented with 10% fetal bovine serum (FBS, Servicebio, G8001-500, Wuhan, China) and 1% penicillin-streptomycin (KeyGen, KGY0023, Nanjing, China). Cell lines were cultured in an incubator with 5% CO_2_ at 37°C.

### RNA extraction, reverse transcription, and quantitative real-time polymerase chain reaction (qRT-PCR)

Total RNA was extracted from cultured cells and tissues using TRIzol Reagent (Sigma, MKCB9720, St. Louis, USA) according to the manufacturer’s instructions. Complementary DNA (cDNA) was reverse transcribed using an RT Reagent Kit (Takara, RR036A, Dalian, China) and RT Master Mix (Takara, RR037A, Dalian, China). qPCR was performed using Premix EX Taq (Takara, RR420A, Dalian, China). All primers used are listed in Supplementary Table 6 and were synthesized by Generay (Shanghai, China). U6 or GAPDH was used as an endogenous control.

### Mimics, inhibitors, small interfering RNAs (siRNAs), vectors, and transfection

miR-125a-5p mimics and inhibitors, small interfering RNAs (siRNAs) for PTPN1 and MAP3K11, and corresponding negative controls were synthesized by GenePharm (Shanghai, China); their sequences are provided in Supplementary Table 7. miR-125a-5p overexpression and knockdown lentiviral vectors were purchased from GenePharm (Shanghai, China). PTPN1 and MAP3K11 cDNA fragments were synthesized by Sangon Biotech (Shanghai, China) and cloned into pCDNA3.1 (+) obtained for a previous study [67]. Mimics, inhibitors, and siRNAs were transfected at 80 nM, and cells were transfected with 2 μg of the vectors per 6 well plate using Lipofectamine2000 (Invitrogen, #1881535, Carlsbad, USA) according to the manufacturer’s instructions.

### Cell counting kit-8 (CCK8) cell proliferation assays

10,000 cells were cultivated in a 96-well plate in a 37°C incubator with 5% CO_2_. 10% CCK8 (Bimake, B34302, TX, USA) was added to each well 24 h, 48 h, 72 h, and 96 h after the cells were transfected. Absorbance optical density (OD) values were measured 1 h later at 450 nm wavelength. Each assay was performed three times.

### Colony formation assay

For the colony formation assay, 300 cells were cultivated in 12-well plates in a 37°C incubator with 5% CO_2_ 24 h after transfection. Ten days later, colonies resulting from the surviving cells were fixed with 95% alcohol (Sangon Biotech, A507050, Shanghai, China), stained with 0.1% crystal violet (Sigma, #SLBP6921V, St. Louis, USA), counted using Image J, and photographed. Each assay was performed three times.

### Dual-luciferase reporter assay

Luciferase reporter constructs containing WT (containing miR-125a-5p binding site) or MT (mutated miR-125a-5p binding site) PTPN1 or MAP3K11 3′-UTR sequences were cloned into the psiCHECK2 luciferase reporter vector. The cells were then co-transfected with a Renilla luciferase vector and psiCHECK2-PTPN1 or psiCHECK2-MAP3K11. Finally, luciferase activity was measured using a Dual Luciferase Reporter Assay Kit (Promega, E1910, Madison, USA) according to the manufacturer’s instructions.

### Western blot analysis

Total protein was extracted from cells after transfection using lysis buffer for western and IP (KeyGen, KGP701, Nanjing, China), and protein concentration was measured using a BCA protein assay kit (Beyotime, P0010, Shanghai, China). Proteinsamples were electrophoresed using SDS-PAGE and then transferred for incubation at 4ºC overnight with the following specific primary antibodies: p53 (ImmunoWay, YT3528, TX, USA), Bax (ImmunoWay, YT0455, TX, USA), Bcl-2 (ImmunoWay, YM3041, TX, USA), active caspase-3 (Abcam, ab32042, Cambridge, UK), PTPN1 (Abcam, ab75856, Cambridge, UK), MAP3K11 (Abcam, ab51068, Cambridge, UK), JNK1/2/3 (Bimake, A5005, TX, USA), c-Jun (Bimake, A5730, TX, USA), and GAPDH (ImmunoWay, YM3445, TX, USA). The samples were then incubated with HRP-conjugated anti-rabbit IgG antibody (CST, #7074, MA, USA) at room temperature for 1 h. Finally, proteins were detected using ECL (KeyGEN, KGP902, Nanjing, China). GAPDH was used as an internal reference. Image J was used for quantification of all Western blot bands.

### Immunofluorescence (IF)

Bel-7404 and SK-Hep1 cells were fixed 24 h after transfection with 4% paraformaldehyde (200 μL) (Servicebio, G1101, Wuhan, China) for 20 min and then washed with 1× PBS (KeyGEN, KGB5001, Nanjing, China) 3 times for 5 min each. The cells were then permeabilized in 3% FBS (Servicebio, G8001-500, Wuhan, China), 1% goat-serum (Beyotime, C0265, Shanghai, China), and 0.1% Triton X-100 (Solarbio, #T8200, Beijing, China) for 1 h at ambient temperature. Next, the cells were incubated with the cleaved caspase substrate antibody (CST, #8698, MA, USA) at 4°C overnight. The next day, the cells were incubated with Cy3-conjugated goat anti-rabbit IgG (Servicebio, GB21303, Wuhan, China) for 1 h at room temperature and stained for 10 min with DAPI (Servicebio, G1012, Wuhan, China). Photographs were taken using an inverted phase contrast microscope (LEICA, DFC550, Germany). Cleaved caspase substrate-positive cells were quantified using Image J.

### Immunohistochemistry (IHC)

Tumor xenografts were fixed in paraformaldehyde (Servicebio, G1101, Wuhan, China) for 12 h and embedded in paraffin. After that, the KeyGEN One-Step IHC Assay (KeyGen, KGOS60-KGO300, Nanjing, China) was used to stain 5-μm-thick paraffin sections, which were then incubated with the following antibodies: Brdu (Servicebio, GB12051, Wuhan, China), Ki-67(ImmunoWay, YT2467, TX, USA), PTPN1(Abcam, ab75856, Cambridge, UK), MAP3K11(Abcam, ab51068, Cambridge, UK), JNK1/2/3 (Bimake, A5005, TX, USA), and c-Jun (Bimake, A5730, TX, USA). The paraffin sections were then photographed.

### Tumor xenografts in animals

A total of 15 4-week-old athymic BALB/c nude mice (SLAC, Shanghai, China) were divided among three groups with five mice in each group. hsa-miR-125a-5p overexpression, knockdown, or control Bel-7404 cells (5 × 10^6^ in 200 μL PBS) were subcutaneously injected into the mice. All cells expressed EGFP. Tumor volumes for each mouse were calculated as follows: tumor volume = length × width × width/2. After 3 weeks, the IVIS Imaging system (Spectral Instruments, USA) was used to observe the tumor xenografts in the mice. The mice were then sacrificed by cervical dislocation under anesthesia with ether, and xenograft tumor tissues were removed for examination.

### Statistical analysis

All results are expressed as mean ± SD. SPSS 20.0 software (SPSS, Chicago, USA) was used for statistical analyses. Student’s t-tests, Chi-square tests, one-and two-way ANOVAs, Kaplan-Meier plots, and Pearson correlation analyses were used for statistical comparisons. *P* < 0.05 was considered statistically significant. Graphs were primarily made using GraphPad Prism 7 (GraphPad, San Diego, USA).

## Supplementary Material

Supplementary Figures

Supplementary Tables
