# Supplementary material for: miR-125a-5p inhibits tumorigenesis in hepatocellular carcinoma
Source: Aging (Albany NY). 2019 Sep 17;11(18):7639–62. doi: 10.18632/aging.102276 (PMC6781988; doi:10.18632/aging.102276)
Supplement: Supplementary Figures [file aging-11-102276-s002.pdf]

SUPPLEMENTARY FIGURES

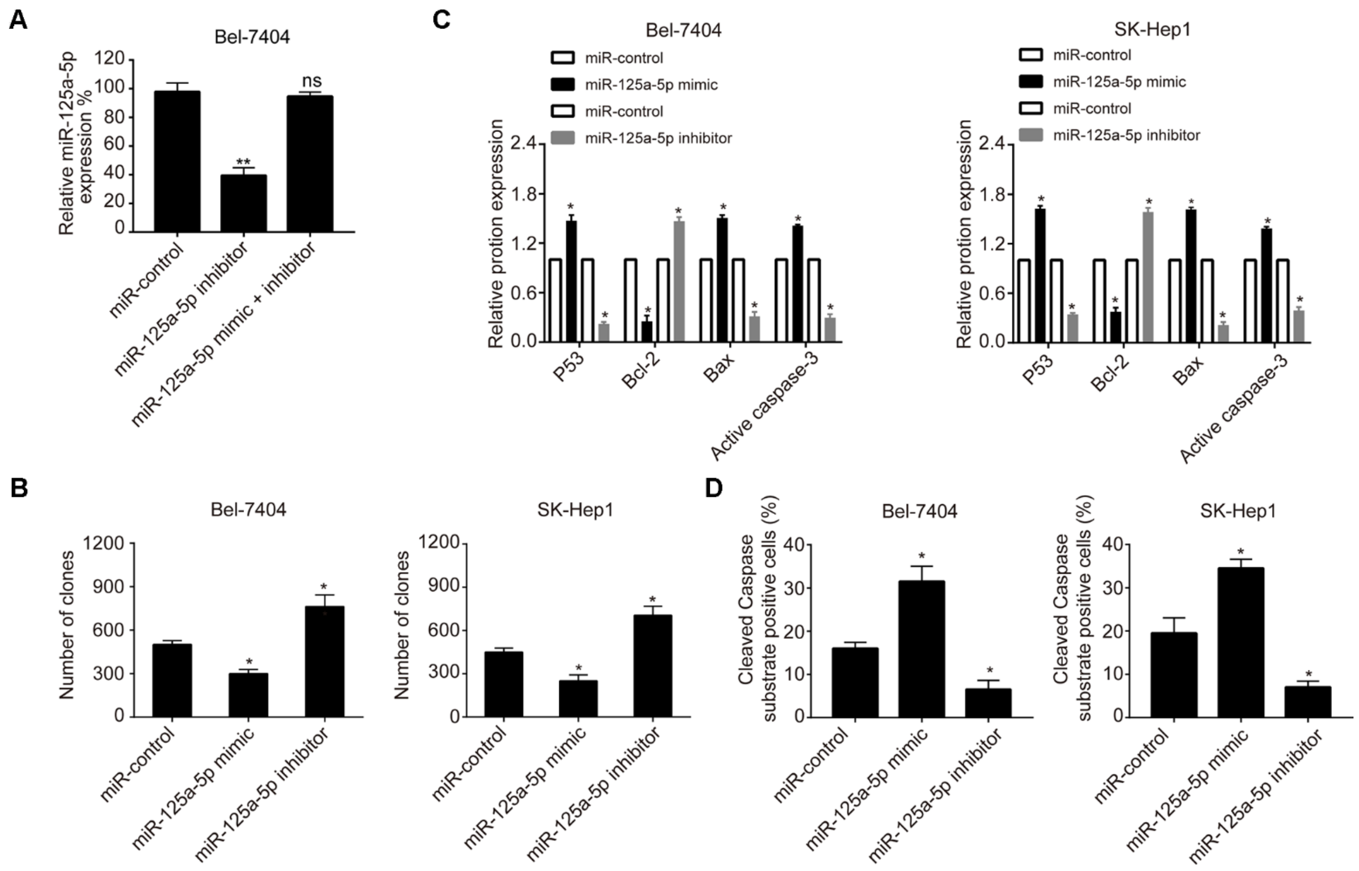

Supplementary Figure 1. miR-125a-5p suppresses proliferation and induces apoptosis in HCC cells *in vitro*. (**A**) Bel-7404 cells were transfected with miR-125a-5p inhibitor and mimic. Student's t-test, mean ± SD, ***P*<0.01, ns: not statistically significant. (**B**) Cell colony numbers in transfected Bel-7404 and SK-Hep1 cells were assessed using Image J. Student's t-test, mean ± SD, **P*<0.05. (**C**) Western blots were used to analyze p53, Bax, Bcl-2, and active caspase-3 expression in transfected Bel-7404 and SK-Hep1 cells. Student's t-test, mean ± SD, **P*<0.05. (**D**) Cleaved caspase substrate-positive cell numbers were assessed after immunofluorescence staining in transfected Bel-7404 and SK-Hep1 cells using Image J. Student's t-test, mean ± SD, **P*<0.05.

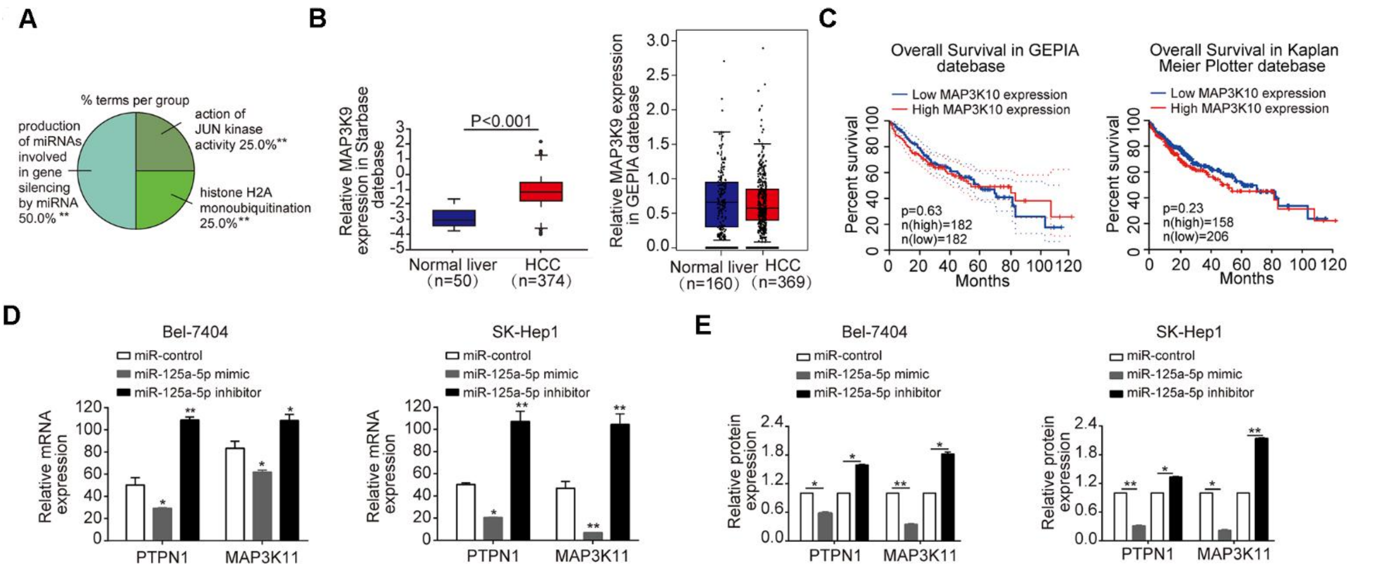

Supplementary Figure 2. PTPN1 and MAP3K11 are direct targets of miR-125a-5p in HCC. (**A**) 131 target genes were analyzed with ClueGO and CluePedia in Cytoscape. (**B**) MAP3K9 was up-regulated in the Starbase database, but down-regulated in the GEPIA database. (**C**) Overall survival of HCC patients was not associated with MAP3K10 expression level in Kaplan Meier Plotter and GEPIA databases. (**D**) PTPN1 and MAP3K11 mRNA expression was detected by qRT-PCR in transfected Bel-7404 and SK-Hep1 cells. One-way ANOVA, mean ± SD, **P*<0.05, ***P*<0.01. (**E**) PTPN1 and MAP3K11 protein expression was detected by Western blot in transfected Bel-7404 and SK-Hep1 cells. Student's t-test, mean ± SD, **P*<0.05, ***P*<0.01.

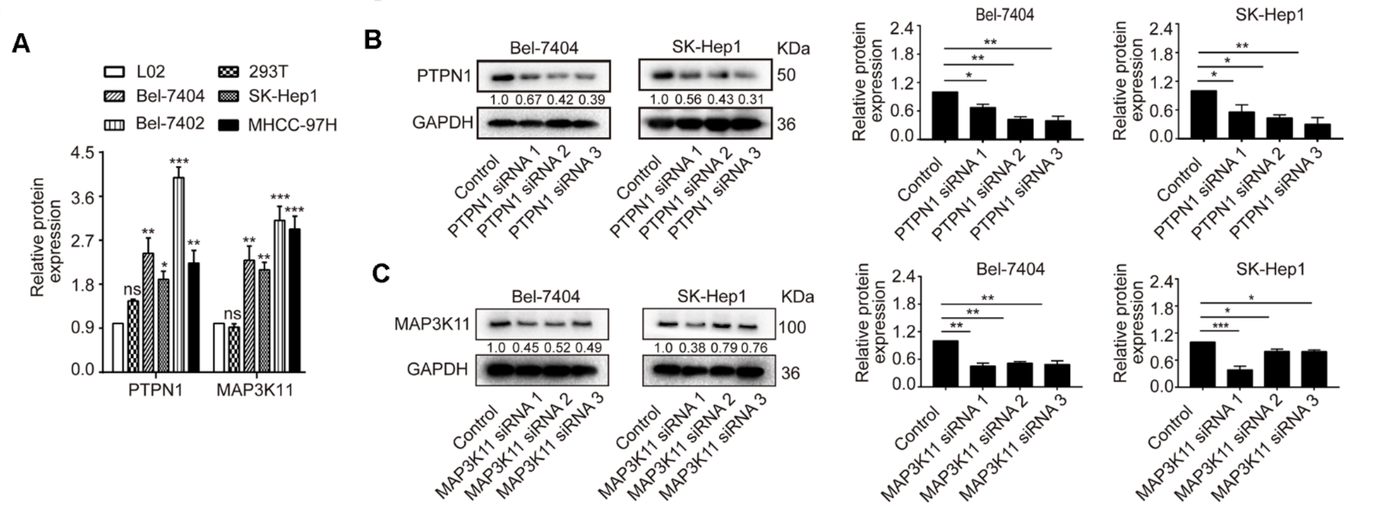

Supplementary Figure 3. PTPN1 and MAP3K11 are up-regulated and negatively correlated with the expression of miR-125a-5p in HCC. (**A**) Western blots indicated that PTPN1 and MAP3K11 were up-regulated in HCC cell lines. One-way ANOVA, mean ± SD, **P*<0.05, ***P*<0.01, ****P*<0.001, ns: not statistically significant. (**B** and **C**) Western blots indicated that PTPN1 and MAP3K11 knockdown decreased PTPN1 and MAP3K11 protein expression. One-way ANOVA, mean ± SD, **P*<0.05, ***P*<0.01.

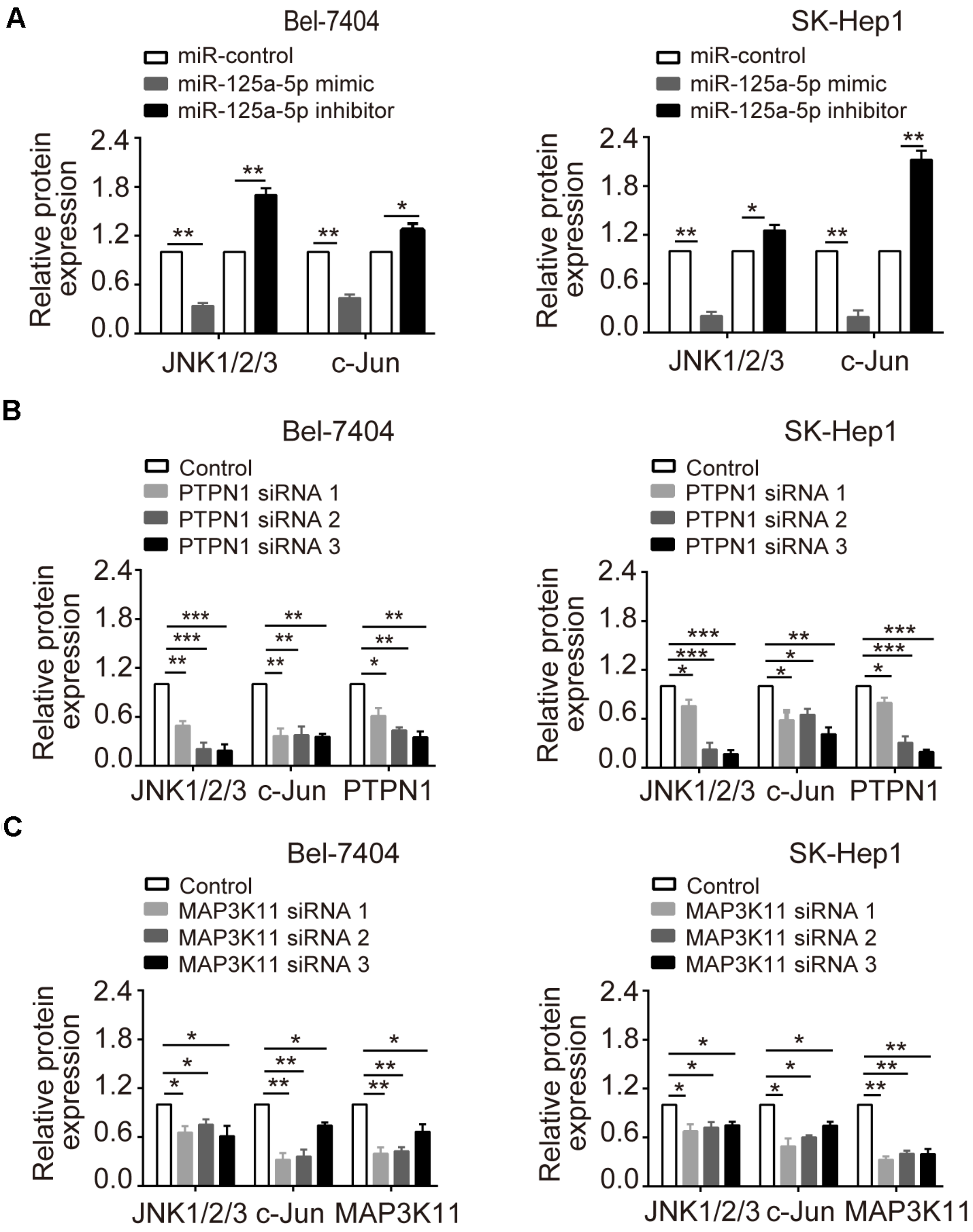

Supplementary Figure 4. miR-125a-5p suppresses PTPN1 and MAP3K11 expression via the MAPK signaling pathway in HCC. (**A**) Western blots showed that miR-125a-5p overexpression reduced, while miR-125a-5p knockdown increased, JNK1/2/3 and c-Jun expression in Bel-7404 and SK-Hep1 cells. Student's t-test, mean ± SD, *P<0.05, **P<0.01. (**B** and **C**) Western blots indicated that PTPN1 and MAP3K11 knockdown decreased JNK1/2/3 and c-Jun expression in Bel-7404 and SK-Hep1 cells. One-way ANOVA, mean ± SD. *P<0.05, **P<0.01 ***P<0.001, ns: not statistically significant.

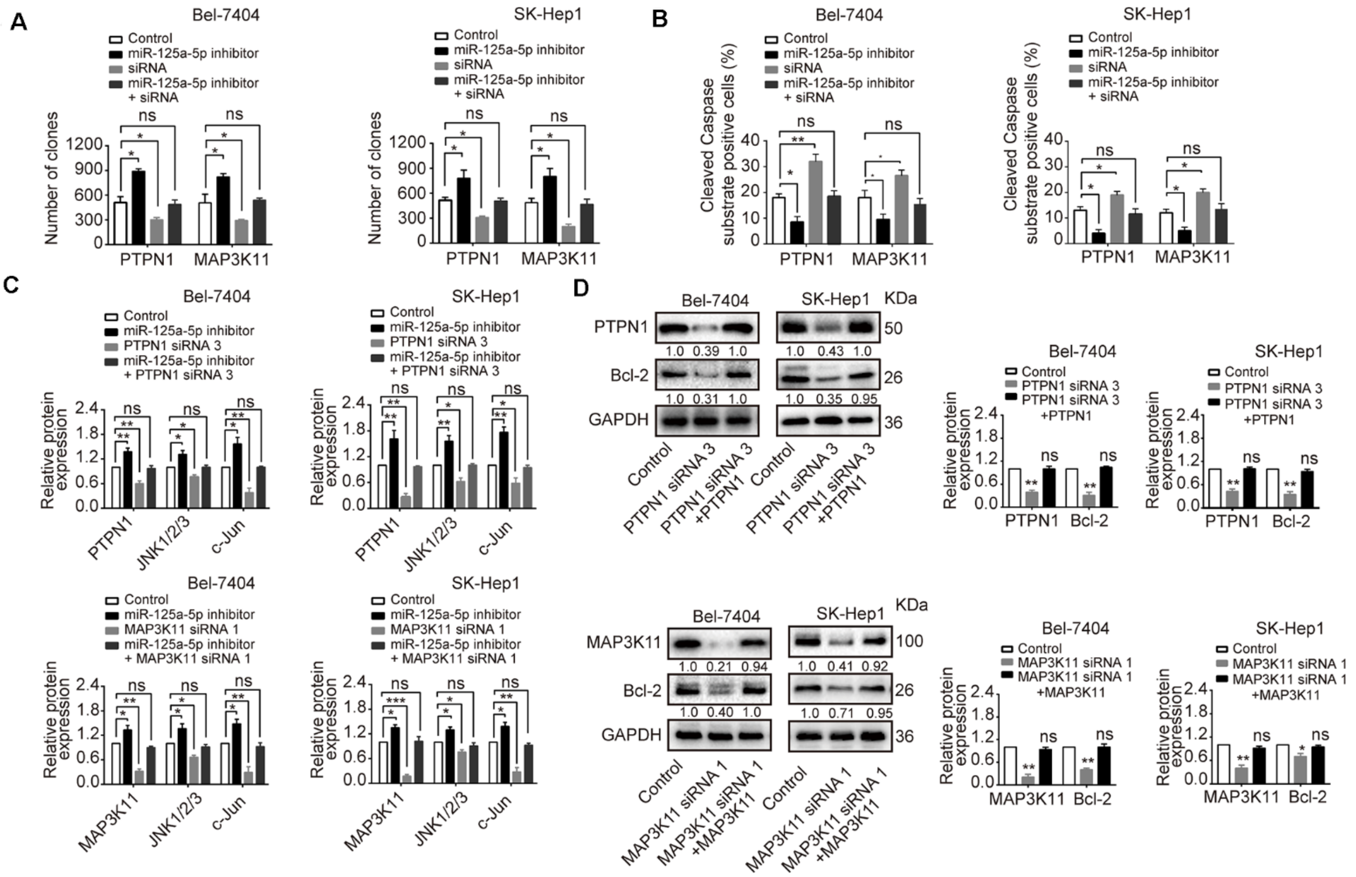

Supplemenary Figure 5. miR-125a-5p suppresses cell proliferation and induces apoptosis in HCC by targeting PTPN1 and MAP3K11 via the MAPK signaling pathway. (**A**) Cell colony numbers were assessed in transfected Bel-7404 and SK-Hep1 cells using Image J. One-way ANOVA, mean ± SD, **P*<0.05, ns: not statistically significant. (**B**) Cleaved caspase substrate-positive cell numbers were assessed after immunofluorescence staining in transfected Bel-7404 and SK-Hep1 cells using Image J. Student's t-test, mean ± SD, **P*<0.05, ***P*<0.01, ns: not statistically significant. (**C**) Western blot experiments were used to analyze PTPN1, MAP3K11, JNK1/2/3, and c-Jun expression in transfected Bel-7404 and SK-Hep1 cells. One-way ANOVA, mean ± SD, **P*<0.05, ***P*<0.01, ****P*<0.001, ns: not statistically significant. (**D**) PTPN1 and MAP3K11 CDS were inserted into pCDNA3.1(+) vectors to confirm acceptable rescue efficiency and to rule out off-target siRNA effects via Western blot. One-way ANOVA, mean ± SD, **P*<0.05, ***P*<0.01, ns: not statistically significant.
