# Supplementary material for: miR-125a-5p inhibits tumorigenesis in hepatocellular carcinoma
Source: Aging (Albany NY). 2019 Sep 17;11(18):7639–62. doi: 10.18632/aging.102276 (PMC6781988; doi:10.18632/aging.102276)
Supplement: Supplementary Tables [file aging-11-102276-s001.pdf]

SUPPLEMENTARY TABLES

Supplementary Table 1. Differentially expressed miRNAs with Fold change, P value, and FDR.

Gene	Fold change	P value	FDR
miR-98	2.024689	0.0014	0.007467
miR-215	2.751503	0.0059	0.00651
miR-101-3p	2.880401	0.0084	0.008671
miR-106a-5p	4.005514	0.001	0.016
miR-125a-3p	5.775092	0.0086	0.0086
miR-125b-5p	2.875402	0.0015	0.006857
miR-125a-5p	9.646382	0.0001	0.0032
miR-126-3p	3.00155	0.001	0.016
miR-127-5p	3.342685	0.0015	0.006857
miR-129-2-3p	2.735577	0.0031	0.005221
miR-139-5p	2.772898	0.0024	0.00512
miR-141-3p	4.608696	0.0036	0.005236
miR-142-5p	3.755943	0.0033	0.00528
miR-146a-5p	2.705622	0.0031	0.005221
miR-15a-5p	4.804255	0.0034	0.005181
miR-181a-5p	2.602305	0.0024	0.00512
miR-181c-3p	3.966543	0.0019	0.005527
miR-186-5p	4.123004	0.0023	0.005257
miR-199a-5p	2.33565	0.0037	0.005148
miR-19b-3p	3.619808	0.0049	0.006272
miR-200a-3p	2.804085	0.0029	0.005459
miR-200b-3p	3.830888	0.0038	0.005067
miR-216a	3.838346	0.0013	0.00832
miR-216b	2.716851	0.0036	0.005236
miR-219-1-3p	3.890149	0.0012	0.0096
miR-223-3p	2.135747	0.002	0.005333
miR-22-3p	2.592771	0.0016	0.0064
miR-26a-5p	2.179431	0.0057	0.006514
miR-26b-5p	2.816377	0.0011	0.011733
miR-27a-3p	2.118625	0.005	0.006154
miR-301a-3p	2.61157	0.0034	0.005181
miR-30a-5p	2.03727	0.0051	0.006044
miR-30c-5p	2.9511	0.0001	0.0032
miR-30e-5p	2.29	0.001	0.016
miR-31-5p	2.004115	0.0012	0.0096
miR-320c	2.351724	0.0011	0.011733
miR-320e	2.490741	0.0013	0.00832
miR-324-5p	2.180824	0.0017	0.006044
miR-338-5p	2.011732	0.002	0.005333
miR-34a-5p	2.067332	0.0016	0.0064
miR-500a-3p	2.001572	0.0015	0.006857
miR-9-3p	3.197425	0.0012	0.0096
miR-96-5p	2.000000	0.0016	0.0064
miR-374a-5p	2.53286	0.0024	0.00512
miR-21-5p	2.28769	0.0018	0.00576
miR-17-3p	2.081494	0.003	0.005333
miR-17-5p	2.179357	0.0016	0.0064
miR-301b	2.169048	0.005	0.006154
miR-495-3p	2.012968	0.0015	0.006857
miR-18a-5p	2.107143	0.0082	0.008747
miR-561-5p	2.818947	0.0018	0.00576
miR-423-5p	2.883028	0.0025	0.005
miR-539-3p	2.389985	0.0017	0.006044
miR-330-5p	2.693243	0.0025	0.005
miR-148b-3p	2.508993	0.0012	0.0096
miR-541-3p	2.358318	0.0011	0.011733

miR-379-3p	2.553738	0.0014	0.007467
miR-382-5p	2.569558	0.001	0.016
miR-411-3p	2.90303	0.0018	0.00576
miR-654-5p	3.112903	0.0012	0.0096
miR-296-5p	3.529891	0.0015	0.006857
miR-524-5p	3.385475	0.0021	0.005169
miR-494-3p	2.635294	0.0015	0.006857
miR-137	2.933628	0.0029	0.005459

Supplementary Table 2. 131 common elements identified in the DIANA-microT miRDB and TargetScan databases.

TRIM71	SLITRK6	TAF9B	SULT4A1	MAMDC2
PCTP	LIN28A	GGT7	CDC42BPG	DENND6A
KIAA1522	SMG1	LRP4	PTPN1	BMPR1B
ENPEP	SLC39A9	PTPN18	LCOR	SUV420H2
MFHAS1	SEMA4F	RYBP	ABCC5	REST
LFNG	MAP3K9	HIF1AN	TMEM120B	TAZ
SEMA4D	EVA1A	RNF168	TP53INP1	SLC6A17
FUT4	NIPAL4	MBD1	NECAB3	WIPF2
MYT1	CORO2A	BDH1	KCTD15	TXNRD1
ZSWIM6	CCNJ	RFXANK	PSMB8	CBX7
SLC46A3	SBNO1	KHNYN	PHC2	FAM134C
CRB2	CYP24A1	TNFSF4	DICER1	SLC4A10
ZSCAN29	IRF4	SCARB2	PPME1	C17orf59
MAP3K11	SYVN1	GRB10	BCAT1	LIPA
RAPGEF5	ETV6	ZNF385A	EIF1AD	KLHL24
FAM169B	KIAA1841	BMF	SLC38A9	GTPBP2
FBXW4	PPAT	ZNF691	ZNRF3	PCSK7
KLF13	IL16	MFN1	KCNH7	KCNA1
ABHD6	SEMA4C	THEMIS2	ABL2	EDEM1
GCNT1	SAMD10	CDR2L	USP37	GGA2
MCL1	KCNS3	MAP3K10	PCGF6	IST1
ESRRA	RHOQ	ZC3H7B	ELOVL6	RREB1
LBH	TRPS1	TLE3	HNRNPUL2	FAM53C
LRFN2	KLHL6	SH3BP5L	NT5DC1	CGN
WARS	SLC7A1	ST6GAL1	TMEM136	MLF2
TGOLN2	IER3IP1	ITGA9	USP46	LIN28B
DAAM1				

Supplementary Table 3. Correlations between PTPN1 and MAP3K11 protein expression and clinicopathologic features.

Feature	Number (n=120)	PTPN1 expression			*P* value	MAP3K11 expression			*P* value
		Low	Medium	High		Low	Medium	High	
Ages									
≥60	26	11	10	5	0.064	7	9	10	0.142
<60	94	23	40	31		13	43	38	
Gender									
Male	94	26	40	28	0.902	8	43	38	0.231
Female	26	8	10	8		7	9	10	
Garde									
1	12	4	2	6	0.835	5	1	6	0.552
2	104	28	47	29		11	51	40	
3	4	2	1	1		1	1	2	
Stage									
I	6	4	1	1	0.008	3	2	1	0.025
II	42	21	11	10		10	21	11	
III	72	9	38	25		7	29	36	

Supplementary Table 4. PTPN1 expression in HCC and normal liver tissues.

	Number	Low	Medium	High	*P* value
Normal	n=10	7	2	1	0.0152
HCC	n=120	34	50	36	

Supplementary Table 5. MAP3K11 expression in HCC and normal liver tissues.

	Number	Low	Medium	High	*P* value
Normal	n=10	5	3	2	0.0382
HCC	n=120	20	52	48	

Supplementary Table 6. Primers used in the qRT-PCR reactions.

Gene	Sequence (5′—3′)
miR-125a-5p-RT	GTCGTATCCAGTGCAGGGTCCGAGGTATTCGCACTGGATACGACTCACAGG
miR-125a-5p-F	ACACTCCAGCTGGGTCCCTGAGACCCTTTAAC
miRNA universal-R	GTGCAGGGTCCGAGGT
U6-F	CTCGCTTCGGCAGCACA
U6-R	AACGCTTCACGAATTTGCGT
PTPN1-F	ACTATACCACATGGCCTGACTT
PTPN1-R	GAAGAAGGGTCTTTCCTCTTGT
MAP3K11-F	CCACCGTGATCTCAAGTCCAAC
MAP3K11-R	AGGCCTTGATAACCTCAGGAGC
GAPDH-F	GGGTGTGAACCATGAGAAGTATG
GAPDH-R	AGTAGAGGCAGGGATGATGTTCT

Supplementary Table 7. Sequences of mimics, inhibitors, and siRNAs.

Name	Sequence (5′—3′)
miR-125a-5p mimic	UCCCUGAGACCCUUUAACCUGUGA
	ACAGGUUAAAGGGUCUCAGGGAUU
miR-125a-5p inhibitor	UCACAGGUUAAAGGGUCUCAGGGA
miR-control	CAGUACUUUUGUGUAGUACAA
PTPN1 siRNA 1	GUCGGAUUAAACUACAUCATT
	UGAUGUAGUUUAAUCCGACTT
PTPN1 siRNA 2	GACCCUUCUUCCGUUGAUATT
	UAUCAACGGAAGAAGGGUCTT
PTPN1 siRNA 3	GAGCCACACAAUGGGAAAUTT
	AUUUCCCAUUGUGUGGCUCTT
MAP3K11 siRNA 1	GCAUCUUCCCGUCCAACUATT
	CAGUUGGACGGGAAGAUGTT
MAP3K11 siRNA 2	CCUAUGGCGUAGCUGUUAATT
	UUAACAGCUACGCCAUAGGTT
MAP3K11 siRNA 3	CAUGGUACCUGGAUUCAGATT
	UCUGAAUCCAGGUACCAUGTT
